# Forskolin Sensitizes Human Acute Myeloid Leukemia Cells to H3K27me2/3 Demethylases GSKJ4 Inhibitor via Protein Kinase A

**DOI:** 10.3389/fphar.2018.00792

**Published:** 2018-07-20

**Authors:** Michela Illiano, Mariarosaria Conte, Luigi Sapio, Angela Nebbioso, Annamaria Spina, Lucia Altucci, Silvio Naviglio

**Affiliations:** ^1^Department of Precision Medicine, School of Medicine, University of Campania “Luigi Vanvitelli”, Naples, Italy; ^2^IRCCS SDN, Napoli Via E. Gianturco, Naples, Italy

**Keywords:** forskolin, leukemia, epigenetics, anticancer therapy, cell death

## Abstract

Acute myeloid leukemia (AML) is an aggressive hematological malignancy occurring very often in older adults, with poor prognosis depending on both rapid disease progression and drug resistance occurrence. Therefore, new therapeutic approaches are demanded. Epigenetic marks play a relevant role in AML. GSKJ4 is a novel inhibitor of the histone demethylases JMJD3 and UTX. To note GSKJ4 has been recently shown to act as a potent small molecule inhibitor of the proliferation in many cancer cell types. On the other hand, forskolin, a natural cAMP raising compound, used for a long time in traditional medicine and considered safe also in recent studies, is emerging as a very interesting molecule for possible use in cancer therapy. Here, we investigate the effects of forskolin on the sensitivity of human leukemia U937 cells to GSKJ4 through flow cytometry-based assays (cell-cycle progression and cell death), cell number counting, and immunoblotting experiments. We provide evidence that forskolin markedly potentiates GSKJ4-induced antiproliferative effects by apoptotic cell death induction, accompanied by a dramatic BCL2 protein down-regulation as well as caspase 3 activation and PARP protein cleavage. Comparable effects are observed with the phosphodiesterase inhibitor IBMX and 8-Br-cAMP analogous, but not by using 8-pCPT-2′-O-Me-cAMP Epac activator. Moreover, the forskolin-induced enhancement of sensitivity to GSKJ4 is counteracted by pre-treatment with Protein Kinase A (PKA) inhibitors. Altogether, our data strongly suggest that forskolin sensitizes U937 cells to GSKJ4 inhibitor via a cAMP/PKA-mediated mechanism. Our findings provide initial evidence of anticancer activity induced by forskolin/GSKJ4 combination in leukemia cells and underline the potential for use of forskolin and GSKJ4 in the development of innovative and effective therapeutic approaches for AML treatment.

## Introduction

Acute myeloid leukemia (AML) is the most common acute leukemia in adults, characterized by the acquisition of chromosomal abnormalities, somatic mutations, and epigenetic changes resulting in a consistent degree of biological and clinical heterogeneity and variable response to treatment (De Kouchkovsky and Abdul-Hay, 2016). Despite the use of intensive chemotherapy regimens and hematopoietic stem cell transplantation, AML is associated with poor prognosis and a 5-year overall survival of <30%, due to rapid disease progression and frequent emergence of drug resistance. AML therapy is itself associated with significant morbidity and mortality, and most surviving patients experience at least one serious treatment-related long-term complication (Godley and Larson, 2008). Therefore, it is critical to develop more effective, less toxic therapies for the treatment of patients with AML. In AML, as well as other hematological and solid malignancies, both epigenetic alterations and genetic lesions contribute to the disease development and progression (Li et al., 2016). Based on the above consideration, in the last few years, diverse epigenetic modulating compounds, able to modify the chromatin status of a cell by targeting chromatin enzymes, have been designed and proposed as beneficial against cancer, including leukemia (Lu and Wang, 2017; Sun et al., 2018). To date, the most common epigenetic compounds are histone deacetylase inhibitors (HDACi), such as MS275 (entinostat) and SAHA (vorinostat). HDACi have entered the clinic and are in advanced phases of clinical trials (West et al., 2014), although the widespread inhibition of the plethora of HDAC-containing complexes represents their major drawback (Mottamal et al., 2015).

Another critical epigenetic mark, playing an important role in many biological processes, is the histone methylation. Of note, four lysine residues (K4, K9, K27, and K36) in the conserved N-terminal tail of the histone are primary targets of specific histone methyltransferases and demethylases (Kampranis and Tsichlis, 2009; Morera et al., 2016). Notably, the tri-methylation on lysine 27 of histone H3 (H3K27me3) induces transcriptional repression, and thereby is associated with controlling gene expression patterns and has been recently involved in several cancer types (Ezponda and Licht, 2014). GSKJ4 is a novel, selective inhibitor of the jumonji family of histone demethylases JMJD3 and UTX, which are the H3K27me2/3-specific demethylases, catalyzing the demethylation of H3K27me2/3. To note, GSKJ4 has been recently shown to act as a potent small molecule inhibitor of the proliferation of cancer cells, including glioma, breast, ovarian, lung cancer cells (Hashizume et al., 2014; Sakaki et al., 2015; Watarai et al., 2016; Dalvi et al., 2017; Sui et al., 2017; Yan et al., 2017). Additionally, in diffuse large B-cell lymphoma GSKJ4 is directly toxic as a single agent and also sensitizes the cells to various clinically approved drugs (Mathur et al., 2017). As far as leukemia is concerned, to our knowledge, the cytotoxic effect of GSKJ4 has only been reported in acute lymphoblastic leukemia (Ntziachristos et al., 2014), whereas the combination of all *trans* retinoic acid (ATRA) and GSKJ4 has been very recently described to significantly increase the cell death compared with ATRA or GSKJ4 treatment alone in PML-RARα-positive leukemic cells (Rejlova et al., 2018). On the other hand, increased intracellular cAMP concentration plays a well-established role in leukemic cell maturation and proliferation (Shayo et al., 2004; Copsel et al., 2011; Murray and Insel, 2013) and, more in general, cAMP, either via protein kinase A (PKA)-dependent or PKA-independent mechanisms, affects numerous cellular functions and is considered very relevant to cancer (Sapio et al., 2014; Kumar et al., 2018).

Very interestingly, the natural cAMP elevating agent compound forskolin is emerging as one of the most promising molecules for potential use in cancer therapy (Sapio et al., 2017; Tripathi and Singh, 2017). Forskolin is a diterpene produced by the roots of the Indian plant *Coleus forskohlii*. Forskolin directly activates the adenylate cyclase enzyme, which generates cAMP from ATP, thus raising intracellular cAMP levels. Notably, the natural compound forskolin has been used for centuries in traditional medicine and its safety has also been documented in conventional modern medicine (Godard et al., 2005; Henderson et al., 2005; Loftus et al., 2015; Genovese et al., 2017; Pateraki et al., 2017).

The present study has been designed to investigate the possible effects of forskolin on the chemo-sensitivity of acute myeloid leukemic cells to GSKJ4 and the underlying molecular mechanisms.

## Materials and Methods

### Antibodies and Chemical Reagents

#### Chemical Reagents

Propidium iodide (PI) (Sigma Life Science, Milan, Italy), bovine serum albumin (BSA) (Microtech, Naples, Italy), 8-Br-cAMP, cAMP analog 8-pCPT-2′-O-Me-cAMP, forskolin, 3-isobutyl-1-methylxanthine (IBMX), and PKA inhibitors KT5720 and H-89 were purchased from Sigma-Aldrich (St. Louis, MO, United States), GSKJ4 (Sigma-Aldrich, St. Louis, MO, United States, #SML0701).

#### Primary Antibodies Used for Immunoblotting

Anti-tubulin antibody (CP06, Oncogene-Calbiochem, La Jolla, CA, United States), anti-ERK (#9102), anti-p-CREB (Ser133, #9198), and anti-CREB (#9197) (Cell Signaling Technology, Danvers, MA, United States), Anti-H3K27me2 (Diagenode, Cat. No. C15410046), and Anti-H3K27me3 (Diagenode, Cat. No. C15410069). All other antibodies were obtained from Santa Cruz Biotechnology (San Diego, CA, United States). Secondary horseradish peroxidase (HRP) conjugated antibodies used for immunoblotting: goat anti-rabbit (GtxRb-003-DHRPX) and goat anti-mouse (GtxMu-003-EHRPX.0.05) (Immunoreagents Inc., Raleigh, NC, United States). Solutions and buffers were prepared with ultra-high quality water.

### Cell Culture and Treatments

U937 and NB-4 cells are leukemic cell lines deriving from a myelo-monocytic leukemia FAB M4, and promyelocytic leukemia FAB M3, respectively, obtained from ATCC and DSMZ, respectively. Cells were kept in RPMI-1640 culture medium containing phenol red (Sigma), supplemented with L-Glutamine (2 mM), 10% of fetal bovine serum (FBS) (Hyclone), penicillin (100 mg/ml), streptomycin (100 mg/ml), and amphotericin B (250 mg/ml) (Sigma, United Kingdom). The cells were incubated at 37°C at a fixed concentration of CO_2_ (5%) and the culture medium was changed every day. At the indicated times and concentrations, treatments with compounds were performed. Forskolin was dissolved in DMSO and added to culture medium in order to obtain the final concentration indicated. Negative control cells were treated with an equal volume of DMSO (<0.1% v/v).

### Cell Proliferation and Cell Cycle Analysis

Colorimetric exclusion: U937 cells (2 × 10^5^ cells/ml) were plated in multiwells and in triplicate. After stimulations at different times and concentrations (as indicated in the text), cells were diluted in the ratio 1:1 in Trypan blue (Sigma) and counted with an optical microscope in order to discriminate dead cells (blue) from living cells, which do not stain.

For cell cycle analyses, the cells were plated (2 × 10^5^ cells/ml) and after stimulation (performed as indicated in the text) were harvested, centrifuged at 1200 rpm for 5 min and resuspended in 500 μl of a hypotonic solution containing 1× PBS, sodium citrate 0.1%, 0.1% NP-40, RNAase A, and 50 mg/ml PI. After 30°C at room temperature in the dark, samples were acquired by FACS-Calibur (BD Bioscences, San Jose, CA, United States) using CellQuest software (BD Biosciences, San Jose, CA, United States). The percentage in different phases of the cell cycle was determined by ModFit LT V3 software (Verity). All experiments were performed in triplicate.

### Cell Death Assay by Propidium Iodide Uptake and Flow Cytometry Analysis

Cell death was measured as previously described (Crowley et al., 2016). Changing of plasma membrane permeability represents the essential condition to allow PI binding to DNA. Due to mechanisms of death (apoptosis, necrosis, autophagy, etc.) plasma membranes generally become permeable. To compare PI uptake, in different cells populations or in the same one, it is used as a method to discriminate dead cells from live cells. Briefly, cells were plated (2 × 10^5^ cells/ml) and grown for 24 h. After that, medium was changed and cells were treated for times, concentrations, and modalities as indicated in the section “Results.” As previously described in the above section, cells were recovered and incubated with PI-FACS buffer containing 0.2 μg/ml of PI in 1× PBS and analyzed by flow cytometry.

### Preparation of Total Cell Lysates

RIPA buffer: 1% NP-40, 0.5% sodium deoxycholate, 0.1% SDS, 10 μg/ml aprotinin, 1 mM leupeptin, and 1 mM phenylmethylsulfonyl fluoride (PMSF). Cells were re-suspended in 3–5 volumes of RIPA buffer and incubated on ice for 1 h. Later, samples were spun-down at 18,000 × g in a table top centrifuge for 15 min at 4°C. Supernatant (SDS total extract) was recovered in order to determine proteins concentration (using Bradford Method) and to prepare samples for immunoblotting (adding Laemmli buffer 4× and boiling).

### Histone Extraction

Cells were harvested and washed twice with cold 1× PBS and lysed in Triton extraction buffer [TEB: PBS containing 0.5% Triton X-100 (v/v), 2 mM PMSF, 0.02% (w/v) NaN 3] at a cellular density of 10^7^ cells/ml for 10 min on ice, with gentle stirring. After a brief centrifugation at 2000 rpm at 4°C, the supernatant was removed and the pellet was washed in half the volume of TEB and centrifuged as before. The pellet was suspended in 0.2 M HCl and acid extraction was left to proceed overnight at 4°C on a rolling table. Next, the samples were centrifuged at 2000 rpm for 10 min at 4°C, the supernatant was removed, and protein content was determined using the Bradford assay as described above.

### Immunodetection of Proteins

Twenty to forty micrograms of proteins from whole extracts were loaded in a polyacrylamide gel (Bio Rad Laboratories, Hercules, CA, United States), separated by SDS–PAGE, and transferred on nitrocellulose membrane (Sigma-Aldrich, St. Louis, MO, United States) using Mini Trans-Blot BioRad (Bio Rad Laboratories, Hercules, CA, United States). Nitrocellulose membranes were blocked with no-fat milk 5% w/v and incubate at +4°C overnight with specific primary antibodies as described in the section “Antibodies and chemical reagents.” The day after nitrocellulose membranes were washed three times per 5 min with TBS Tween-20 (Thermo Fisher Scientific, Waltham, MA, United States) and incubate at room temperature for 1 h with goat anti-rabbit or anti-mouse antibodies conjugated with HRP, used as a detection system (ECL) according to the manufacturer’s instructions (Amersham Biosciences, United Kingdom).

### Statistical Analysis

Data were presented as the mean ± SD of biological replicates. Differences in mean between different groups were calculated using analysis of variance (ANOVA) plus Student’s *t*-test. *P*-values of less than 0.05 were recognized as significant.

## Results

### Forskolin Sensitizes Human Leukemia U937 Cells to GSKJ4

In the present study, we employed the human leukemia U937 cell line as a well-established and widely used model system of human leukemic cells (Shayo et al., 2004; Copsel et al., 2011). Throughout our *in vitro* experiments, we used 10 μM final concentration of forskolin (not toxic, submaximal dose), in agreement with several studies regarding *in vitro* effects by forskolin, ranging from 1 up to 100 μM (Shayo et al., 2004; Dong et al., 2015; Follin-Arbelet et al., 2015; Park and Juhnn, 2016; Pattabiraman et al., 2016; Xiao and Kan, 2017), and a spectrum of final concentration of GSKJ4 up to 10 μM, according to previous findings (Hashizume et al., 2014; Ntziachristos et al., 2014; Sakaki et al., 2015; Watarai et al., 2016; Dalvi et al., 2017; Mathur et al., 2017; Sui et al., 2017; Yan et al., 2017; Rejlova et al., 2018).

**FIGURE 1:**
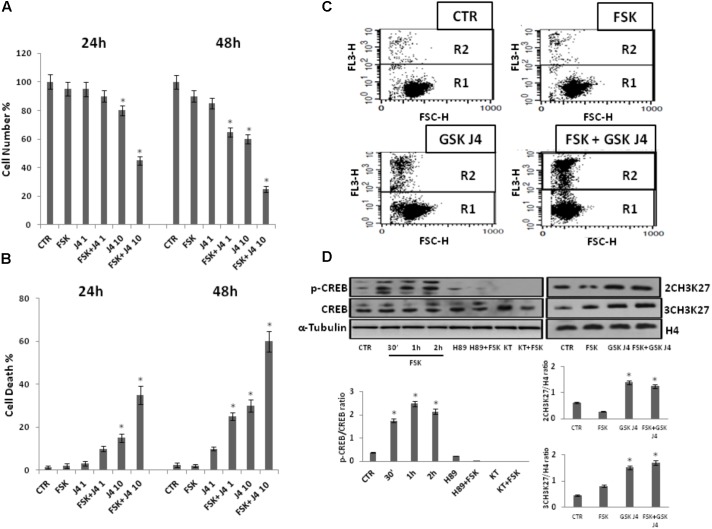
Effects of forskolin on the sensitivity of U937 cells to GSKJ4. Panels **A**, **B**: U937 cells were treated or not with 1 and 10 μM GSKJ4 alone or in combination with 10 μM forskolin for 24 and 48 h. Subsequently, the cell number was recorded (panel **A**) and PI cell death assay was performed (panel **B**). Data represent the average of four independent experiments. The means and SD are shown. ^∗^*P* < 0.05, compared to control untreated cells. Panel **C**: Representative qualitative data of a typical cell death experiment with 10 μM GSKJ4 alone or in combination with forskolin for 24 h are shown. Panel **D**: U937 cells were treated or not with 10 μM forskolin and 10 μM GSKJ4 alone or in combination for the indicated times. The phosphorylation status and levels of CREB protein in the absence and presence of H89 and KT5720 PKA inhibitors, as well as the level of H3K27me2/3 were assessed by western blotting from 30 μg of protein in total cell extract and 1 μg of protein in histone extract, respectively. The image shown is representative of three different experiments with similar results. In the lower part, graphs showing the densitometric intensity of the indicated bands ratio are reported. The intensities of signals are expressed as arbitrary units. ^∗^*P* < 0.05, compared to control untreated cells.

Recently, GSKJ4 has been shown to inhibit the proliferation of many types of cancer cells at micromolar concentrations, with low/no toxicity to normal cells (Hashizume et al., 2014; Ntziachristos et al., 2014; Sakaki et al., 2015; Watarai et al., 2016; Dalvi et al., 2017; Mathur et al., 2017; Sui et al., 2017; Yan et al., 2017). Firstly, we investigated whether GSKJ4 could have an antiproliferative action also on U937 cells and whether forskolin could affect such possible action. To this purpose, we treated U937 cells with two concentrations, 1 and 10 μM, of GSKJ4, in the presence or absence of 10 μM forskolin, for 24 and 48 h. After treatments, direct cell number counting and PI uptake cell death assays were performed (**Figure 1**).

Notably, **Figure 1** shows that proliferation of U937 cells was inhibited by GSKJ4 in dose- and time-dependent manner. More in details, at the dose of 10 μM GSKJ4, the growth inhibition is clear and increases from ≈15% at 24 h to ≈35% at 48 h, whereas 1 μM GSKJ4 has an antiproliferative effect of ≈15% evident only after 48 h of treatment. Interestingly, we found that the co-treatment with forskolin clearly enhanced the anti-proliferative effects of GSKJ4 in all the combinations. To note, at low dose of 1 μM GSKJ4, the growth inhibition increases from ≈15 to ≈30% at 48 h in the presence of forskolin. Impressively, the 10 μM GSKJ4/forskolin combination, already at 24 h, results in a growth inhibition of more than 40% compared with ≈15% obtained with 10 μM GSKJ4 alone.

Moreover, in **Figure 1** it is also observed that forskolin, according to previous findings, does not have toxic effects in U937 cells (Shayo et al., 2004; Copsel et al., 2011). To increase specificity and reliability of our results, we also tested whether forskolin could potentiate the antiproliferative effects of GSKJ4 also on NB-4 cells. Interestingly, that was the case (Supplementary Data, **Supplementary Figure S1**). In addition, we also verified if, in our settings, forskolin and GSKJ4 were effectively acting as PKA activating agent and H3K27me2/3 demethylases inhibitor, respectively (**Figure 1D**).

As shown in **Figure 1D**, in agreement with previous findings, CREB protein, a major substrate of PKA, is strongly phosphorylated in response to forskolin (Naviglio et al., 2010; Li et al., 2017). Notably, such phosphorylation is completely prevented by pretreatment of U937 cells with PKA inhibitors. Thereafter, western blotting was also applied to examine the content of H3K27me2/3. To note, as expected, the global level of H3K27me2/3 was clearly increased in response to GSKJ4 demethylases inhibitor (**Figure 1D**)

Overall, the above data indicate that the PKA activating agent forskolin greatly enhances the sensitivity of U937 cells to H3K27me2/3 demethylases inhibitor GSKJ4.

### Forskolin Enhances the Sensitivity of U937 to GSKJ4 by Inducing Apoptotic Cell Death

**FIGURE 2:**
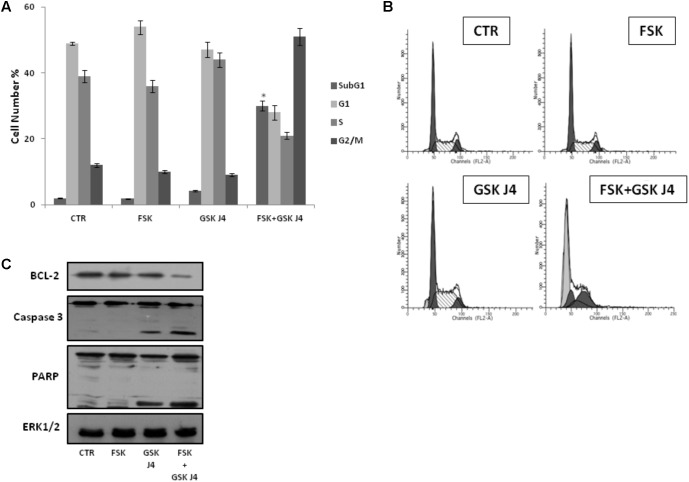
Effects of forskolin, GSKJ4, and forskolin/GSKJ4 combination on the distribution of U937 cells in cell cycle and sub-G1 phases, and on some relevant apoptotic proteins. Treatments with 10 μM forskolin, 10 μM GSKJ4, and forskolin/GSKJ4 combination were carried out for 24 h. Subsequently, sub-G1 and Cell Cycle Phases (panels **A**, **B**) were evaluated by Flow Cytometry. In panel **A**, quantitative data from four independent experiments indicating the percentage of sub-G1, G1, S, and G2/M cells are shown. The means and SD are shown. ^∗^*P* < 0.05, compared to control untreated cells. In panel **B**, representative qualitative data of a typical experiment are shown. In panel **C**, the levels of the indicated proteins were assessed by western blotting from 20 μg of protein in total cell extract. The image shown is representative of three different experiments with similar results.

To further explore the enhancement of GSKJ4-induced anti-proliferative effects by forskolin, U937 cells were exposed or not (control) to 10 μM GSKJ4 in the presence or absence of 10 μM forskolin for 24 h. Subsequently, the distribution of U937 cells in the cell cycle phases was evaluated by flow cytometric analysis of PI-stained cells (**Figures 2A,B**). The proportion of cells with hypodiploid DNA content (sub-G1 population), characteristic of cells having undergone DNA fragmentation, a biochemical hallmark of apoptosis, was also monitored. **Figures 2A,B** show that U937 cells treated with GSKJ4 or forskolin alone do not appear differently distributed in the cell cycle phases, with only insignificant variations, compared to control untreated ones. Interestingly, in **Figure 2** it is also shown that no appearance of a sub-G1 population in response to forskolin occurs, whereas a little percentage (≈5%) of sub-G1 cells is evident upon treatment with GSKJ4 inhibitor. In addition, consistent with the PI uptake cell death assay (above results, **Figure 1**), a large amount of sub-G1 cells (≈30%) was observed in response to forskolin/GSKJ4 combination, strongly suggesting apoptosis induction (**Figures 2A,B**). To further prove the apoptosis occurrence upon forskolin/GSKJ4 combination, we also assessed the levels of some proteins relevantly involved in apoptosis in response to our treatments. To this purpose, U937 cells were exposed for 24 h to forskolin, GSKJ4, and forskolin/GSKJ4 combination. Thereafter, cell extracts were analyzed by western blotting to examine the levels of anti-apoptotic BCL-2 and of caspase 3 and PARP proteins (**Figure 2C**). According to sub-G1 appearance, the level of anti-apoptotic BCL-2 protein was dramatically decreased in response to forskolin/GSKJ4 combination, consistent with cell death by apoptosis. This was further confirmed by examining activation of the terminal caspase-3, executioner of apoptosis, and cleavage of PARP, a known target for apoptosis-associated caspase cleavage. Indeed, **Figure 2C** shows also a decrease of the uncleaved isoform of caspase-3 and the appearance of the cleaved isoform of caspase-3 in U937 cells treated with forskolin/GSKJ4 combination, suggesting the increase of its activity. Moreover, the pattern of the PARP processing proceeds in parallel with that of caspase-3 cleavage (**Figure 2C**).

Altogether, the above data strongly suggest that forskolin enhances the sensitivity of U937 cells to GSKJ4, mainly by inducing apoptotic cell death.

### Forskolin Enhances the Sensitivity of U937 Cells to GSKJ4 via a cAMP/PKA-Dependent Pathway

**FIGURE 3:**
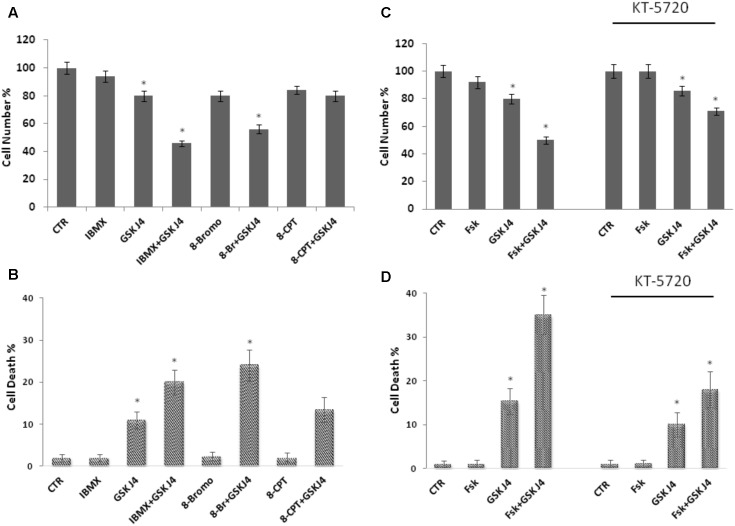
Effects of IBMX, 8-Br-cAMP, and 8-pCPT-2′-O-Me-cAMP on GSKJ4-induced cytotoxicity, and of the PKA inhibitor KT5720 on the proliferation of U937 cells in response to forskolin, GSKJ4, and forskolin/GSKJ4 combination. Panels **A**, **B**: U937 cells were treated or not for 24 h with GSKJ4 10 μM in the absence or presence of 2 mM IBMX, 25 μM 8-pCPT-Me-cAMP, 25 μM 8-Br-cAMP. Panels **C**, **D**: U937 cells were treated or not for 24 h with GSKJ4 10 μM in the absence or presence of 10 μM forskolin and in the absence or presence of 10 μm PKA inhibitor KT5720. Subsequently, the cell number was recorded (panels **A**, **C**) and PI cell death assay was performed (panels **B**, **D**). Data represent the average of three independent experiments. The means and SD are shown. ^∗^*P* < 0.05, compared to control untreated cells.

Forskolin is a direct activator of the adenylate cyclase enzyme and a largely known cAMP elevating compound. However, forskolin can affect other cellular activities (Wong et al., 1993; Yajima et al., 1995). To verify whether the above phenotypes of forskolin could be effectively attributed to the cAMP increase, we also evaluated the effects of another cAMP elevating agent on the GSKJ4-induced cytotoxicity. To this purpose, we treated or not (control) U937 cells for 24 h with GSKJ4 10 μM in the absence or presence of 2 mM IBMX, a broad-spectrum phosphodiesterase inhibitor (**Figures 3A,B**). After treatments, direct cell number counting and PI uptake cell death assays were performed. In **Figures 3A,B** it is shown that the proliferation of U937 cells was not obviously affected by IBMX, when used alone. Interestingly, we found that the co-treatment with cAMP elevating agent IBMX enhanced the anti-proliferative effects of GSKJ4, at similar extent of forskolin (**Figures 3A,B**). In addition, we included in these experiments 8-Br-cAMP, a common analog of cAMP, and 8-pCPT-2′-O-Me-cAMP, a cAMP analog, which specifically activates Epac and not PKA (Grandoch et al., 2009; García-Morales et al., 2017). Similarly to forskolin, 8-Br-cAMP, which is expected to activate both PKA and Epac, potentiated the GSKJ4-induced cytotoxicity. In contrast, the Epac activator 8-pCPT-2′-O-Me-cAMP had only a minimal impact on GSKJ4 effect. Overall, the above data indicate that forskolin potentiates the sensitivity of U937 cells to GSKJ4 via cAMP elevation and that, very likely, PKA might be involved in.

To further investigate the role of PKA on the enhancement by forskolin of the GSKJ4-induced anti-proliferative effects in U937 cells, we used the KT5720 compound, which is a cell-permeable, selective, and potent PKA inhibitor (Davies et al., 2000; El-Zohairy et al., 2015). We tested its effects on the proliferation of U937 cells in response to forskolin, GSKJ4, and forskolin/GSKJ4 combination (**Figures 3C,D**). We found that the PKA inhibitor has a minimal effect on cell proliferation by itself. Importantly, in the presence of KT5720 inhibitor, the enhancement by forskolin of the GSKJ4-induced cytotoxicity is clearly counteracted (**Figures 3C,D**). Similar results were obtained also with PKA inhibitor H-89 (Supplementary Data, **Supplementary Figure S2**).

Overall, the above data strongly suggest that, although other cAMP-dependent mechanisms cannot finally be ruled out, forskolin enhances the sensitivity of U937 cells to GSKJ4 mainly via a cAMP/PKA-dependent pathway.

## Discussion

Currently, new efficacious therapeutic approaches are needed for the treatment of AML. Combination chemotherapy, which promises higher efficacy and lower toxicity of clinical anticancer drugs, is consistently being investigated.

The H3K27me2/3-specific demethylases GSKJ4 inhibitor is emerging as very interesting small epigenetic modifier showing anticancer activity in various cancer cells. The natural cAMP elevating agent forskolin has been proposed as a very promising compound for possible use in cancer therapy. Interestingly, forskolin has recently been shown to increase sensitivity to conventional antitumor drugs in myeloma, colorectal, pancreatic, and triple negative breast cancer cells (Cristóbal et al., 2014; Follin-Arbelet et al., 2015; Illiano et al., 2017, 2018). Consistently, we describe how forskolin in combinatorial treatments markedly enhances the toxicity of the epigenetic agent GSKJ4 in leukemia cells, inducing apoptotic cell death paralleled by a strong reduction of BCL-2 protein levels, via a mechanism dependent on PKA activity.

Depending on the cell type and nature of death-inducing signal, controversial effects of cAMP concerning cell death and potentiation of chemotherapeutic drugs have been described. While the above studies clearly indicate that forskolin, as well as other cAMP elevating agents, can cause cell death or potentiate its induction by other compounds, some studies provide evidence that cAMP elevation decreases chemotherapeutic-induced cell death in cancer cells (Insel et al., 2012).

Strikingly, in leukemia cells, cyclic AMP elevation, that does inhibit their proliferation and enhance their differentiation, has been shown to confer drug resistance and to protect cells against DNA damaging agents-induced apoptosis via PKA-mediated (inactivating) phosphorylation of Ser118 of pro-apoptotic Bad and (activating) phosphorylation of Ser133 of the oncogene CREB (Gausdal et al., 2013; Xiao and Kan, 2017). In apparent contrast to such findings, in the current study we describe that the cAMP elevating agent forskolin, in a PKA-mediated mechanism, causes cell death induction accompanied by BCL-2 down regulation when used in combination with the H3K27me2/3-specific demethylases GSKJ4 inhibitor in U937 leukemia cells. However, we do know that the molecular mechanisms underlying the cAMP/forskolin-induced increase of sensitivity of leukemia cells to GSKJ4 inhibitor are just becoming to be comprehended and need to be exhaustively investigated further.

Here, in agreement with previous findings, we provide initial evidence that GSKJ4 compound increases the global level of H3K27me2/3 methylation and acts as a small molecule inhibitor of the proliferation of myeloid leukemia cells, mechanistically suggesting that GSKJ4 might have anticancer activity by inhibiting demethylases JMJD3 and UTX. Elevation in the global level of H3K27me3 by JMJD3 and UTX demethylases inhibition might lead to the silencing of transcription factors, such as CREB, as well as of other oncogenes, relevantly involved in leukemia (Mitton et al., 2016; Castelli et al., 2017). Understanding the mechanisms by which GSKJ4 inhibits leukemia cell proliferation is an important issue and we are planning to investigate it in future studies. So far, whatever the exact mechanism(s), here we address that exogenous addition of the cAMP elevating agent forskolin enhances the antiproliferative effects of GSKJ4 epigenetic compound via PKA in U937 leukemia cells. Importantly, our results confirm the cytotoxic action of GSKJ4 inhibitor on leukemia cells reinforcing the evidence of GSKJ4 as a small molecule inhibitor with anticancer potential and they demonstrate that co-administration of forskolin significantly increases the anti-cancer activity of GSKJ4 *in vitro*. Remarkably, forskolin is an herbal ingredient that is already on market today as a component of weight-loss dietary supplements (Kanne et al., 2015; Pateraki et al., 2017). Clinical studies, in which oral administration of forskolin (up to 50 mg/day) is used as a weight-loss agent, clearly report an effect on body fat management without no relevant side effects (Godard et al., 2005; Henderson et al., 2005; Loftus et al., 2015; Genovese et al., 2017).

According to our findings, further studies exploring forskolin as a GSKJ4 sensitizer/adjuvant *in vivo* and forskolin/GSKJ4 combination as “communicative reprogramming therapy” are encouraged.

Notably, “Anakoinosis” is a novel concept, describing communicative reprogramming of cancer microenvironment as a way to correct cancer tissue homeostasis. Our opinion is that our study fits in this concept and increases the molecular evidences showing how specific signaling pathways (such as PKA) lead to alteration of key cancer features.

## Conclusion

Our findings provide initial evidence of anticancer activity induced by forskolin/GSKJ4 combination *in vitro* in leukemia cells and underline the potential for use of forskolin and GSKJ4 in the development of innovative and effective therapeutic approaches for AML treatment.

## Author Contributions

MI and MC performed the flow cytometry-based assays of cell-cycle progression and cell death. MI performed the immunoblotting experiments. MC performed direct cell number counting. LS performed the statistical analysis and helped in the preparation of the figures. AN and AS helped to design the study and to draft the manuscript. LA and SN designed and conceived the study and drafted the manuscript. All authors read and approved the final manuscript.

## Conflict of Interest Statement

The authors declare that the research was conducted in the absence of any commercial or financial relationships that could be construed as a potential conflict of interest.

## Supplementary Material

FIGURE S1 Effects of forskolin on the sensitivity of NB-4 cells to GSKJ4. NB-4 cells were treated or not with 1 and 10 μM GSKJ4 alone or in combination with 10 μM forskolin for 24 h. Subsequently, the cell number was recorded **(A)** and PI cell death assay was performed **(B)**. Data represent the average of three independent experiments. The means and SD are shown. ^∗^*P* < 0.05, compared to control untreated cells.

FIGURE S2 Effects of the PKA inhibitor H89 on the proliferation of U937 cells in response to forskolin, GSKJ4, and forskolin/GSKJ4 combination. U937 cells were treated or not for 24 h with GSKJ4 10 μM in the absence or presence of 10 μM forskolin and in the absence or presence of 10 μm PKA inhibitor H89. Subsequently, the cell number was recorded **(A)** and PI cell death assay was performed **(B)**. Data represent the average of three independent experiments. The means and SD are shown. ^∗^*P* < 0.05, compared to control untreated cells.
