# Supplementary figures and images for: Forskolin Sensitizes Human Acute Myeloid Leukemia Cells to H3K27me2/3 Demethylases GSKJ4 Inhibitor via Protein Kinase A
Source: Front Pharmacol. 2018 Jul 20;9:792. doi: 10.3389/fphar.2018.00792 (PMC6063003; doi:10.3389/fphar.2018.00792)

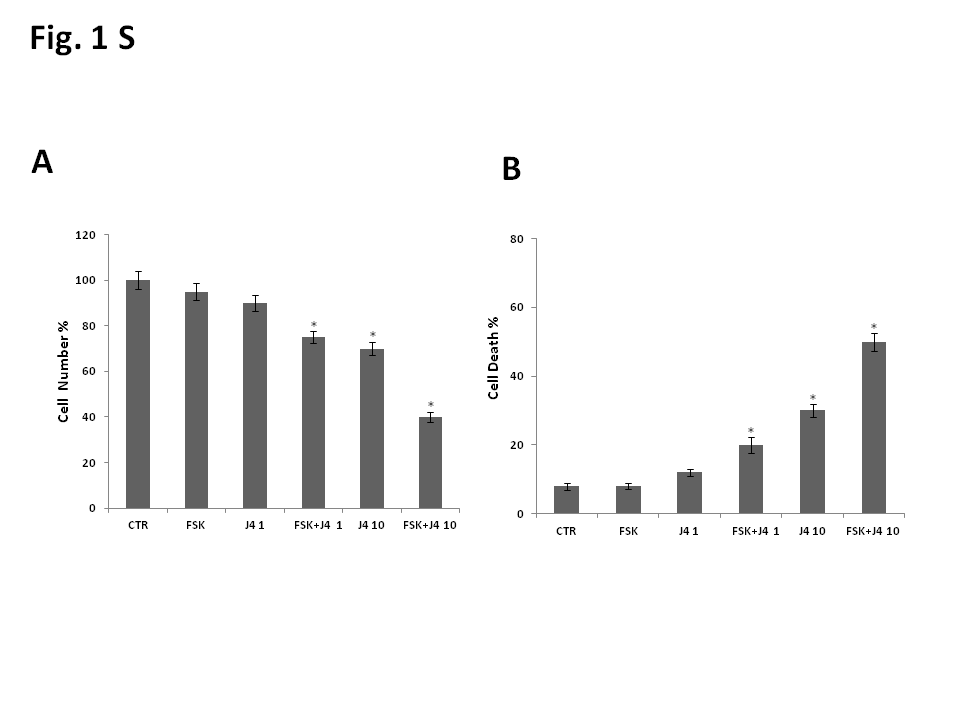

Supplement: FIGURE S1 — Effects of forskolin on the sensitivity of NB-4 cells to GSKJ4. NB-4 cells were treated or not with 1 and 10 μM GSKJ4 alone or in combination with 10 μM forskolin for 24 h. Subsequently, the cell number was recorded (A) and PI cell death assay was performed (B). Data represent the average of three independent experiments. The means and SD are shown. ∗P < 0.05, compared to control untreated cells. [file Image_1.TIF]

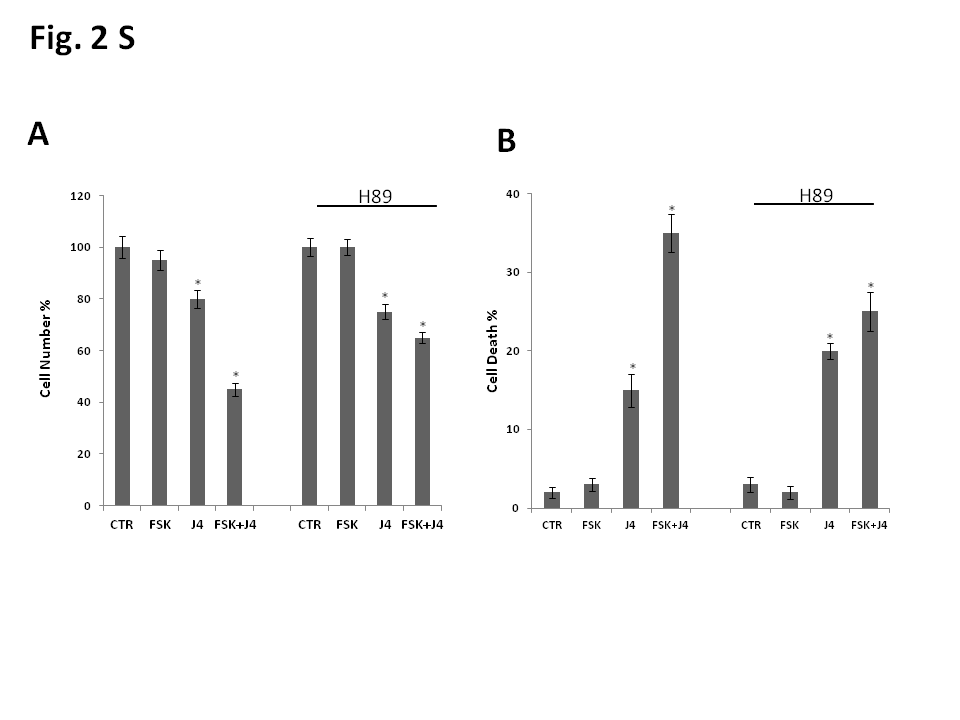

Supplement: FIGURE S2 — Effects of the PKA inhibitor H89 on the proliferation of U937 cells in response to forskolin, GSKJ4, and forskolin/GSKJ4 combination. U937 cells were treated or not for 24 h with GSKJ4 10 μM in the absence or presence of 10 μM forskolin and in the absence or presence of 10 μm PKA inhibitor H89. Subsequently, the cell number was recorded (A) and PI cell death assay was performed (B). Data represent the average of three independent experiments. The means and SD are shown. ∗P < 0.05, compared to control untreated cells. [file Image_2.TIF]

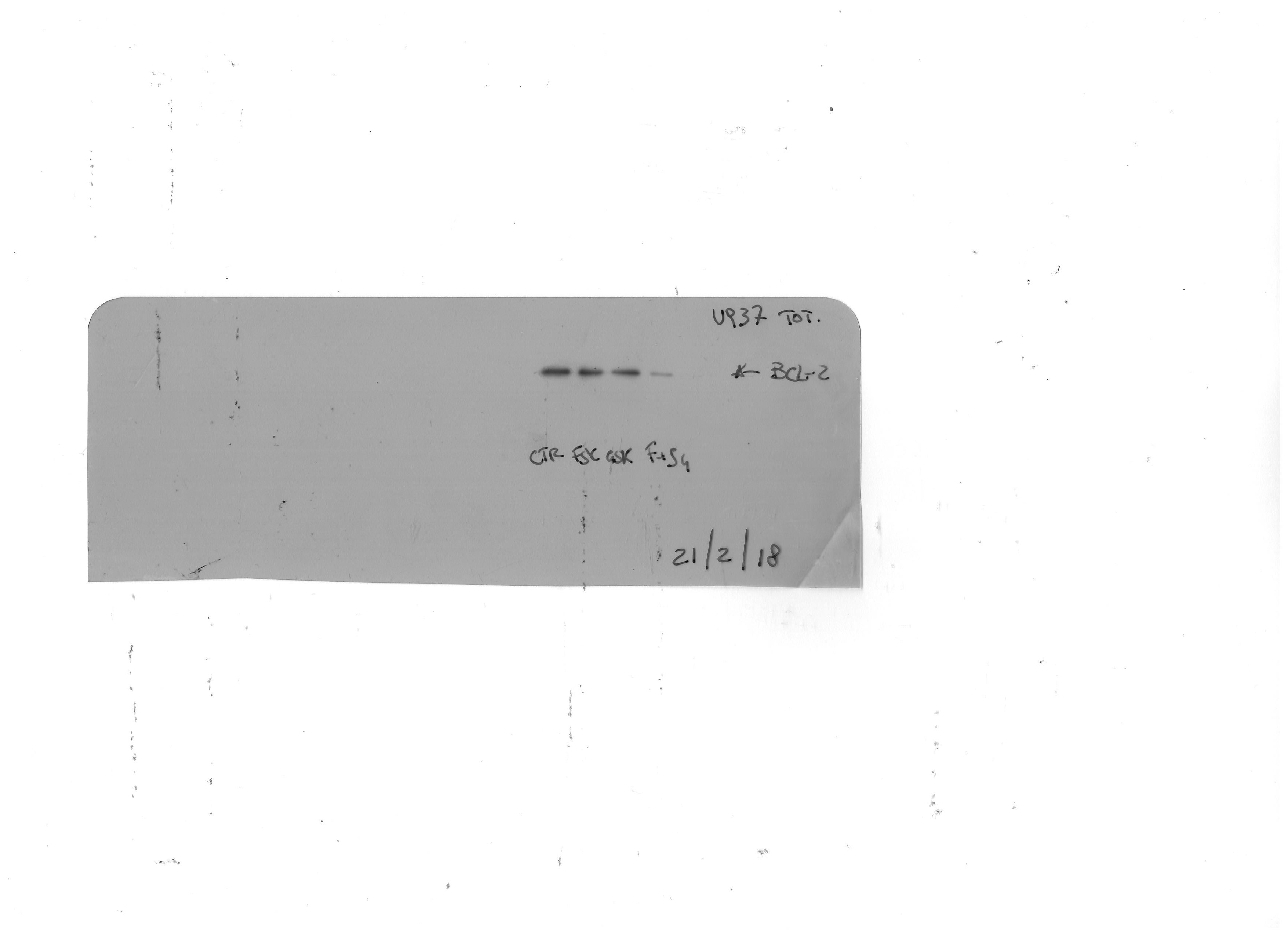

Supplement: Supplementary file 3 [file Data_Sheet_1.ZIP › 382492 _supp images/bcl 2_21.02.jpg]

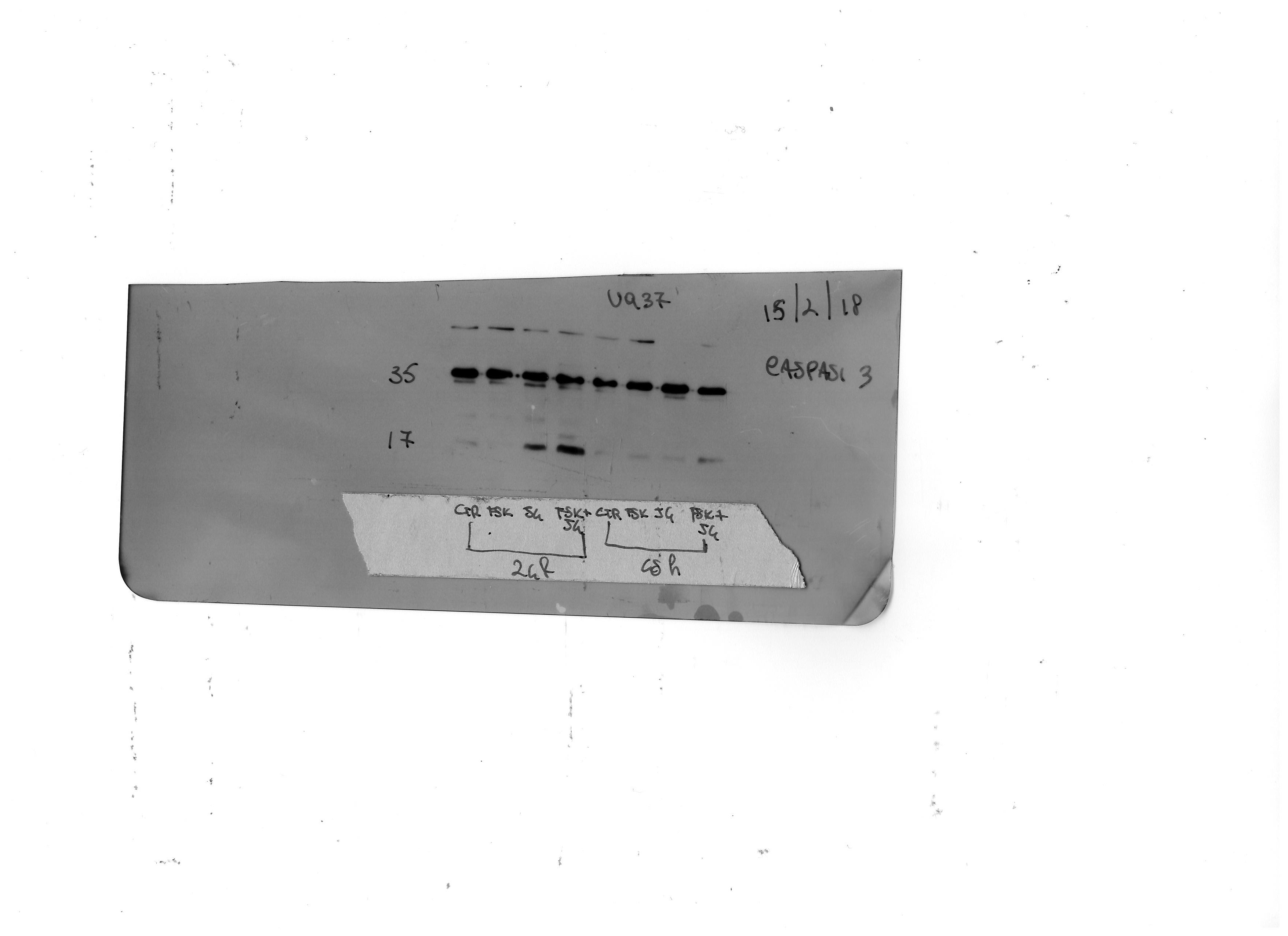

Supplement: Supplementary file 3 [file Data_Sheet_1.ZIP › 382492 _supp images/caspase3 15.02.jpg]

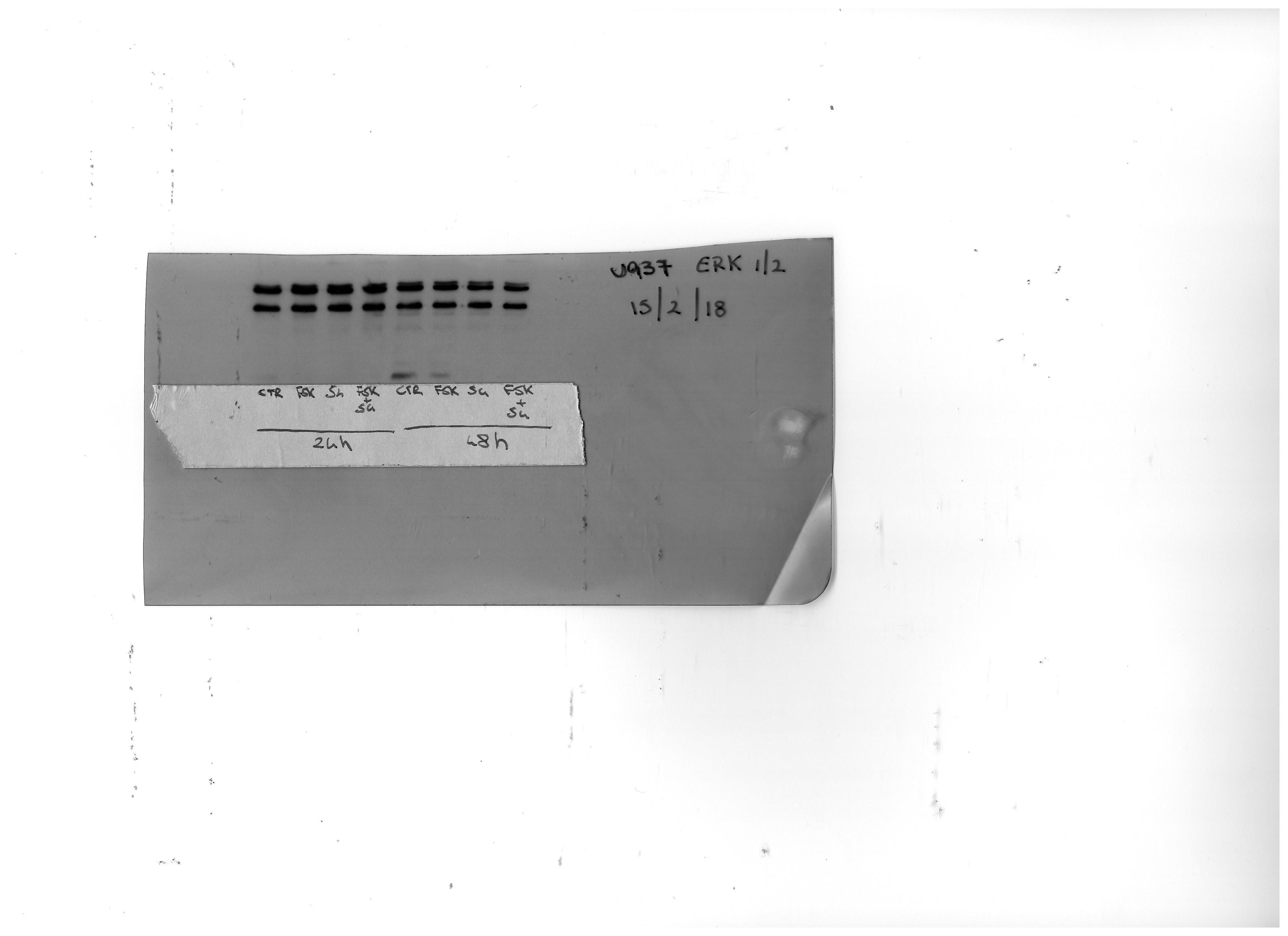

Supplement: Supplementary file 3 [file Data_Sheet_1.ZIP › 382492 _supp images/erk 15.02.jpg]

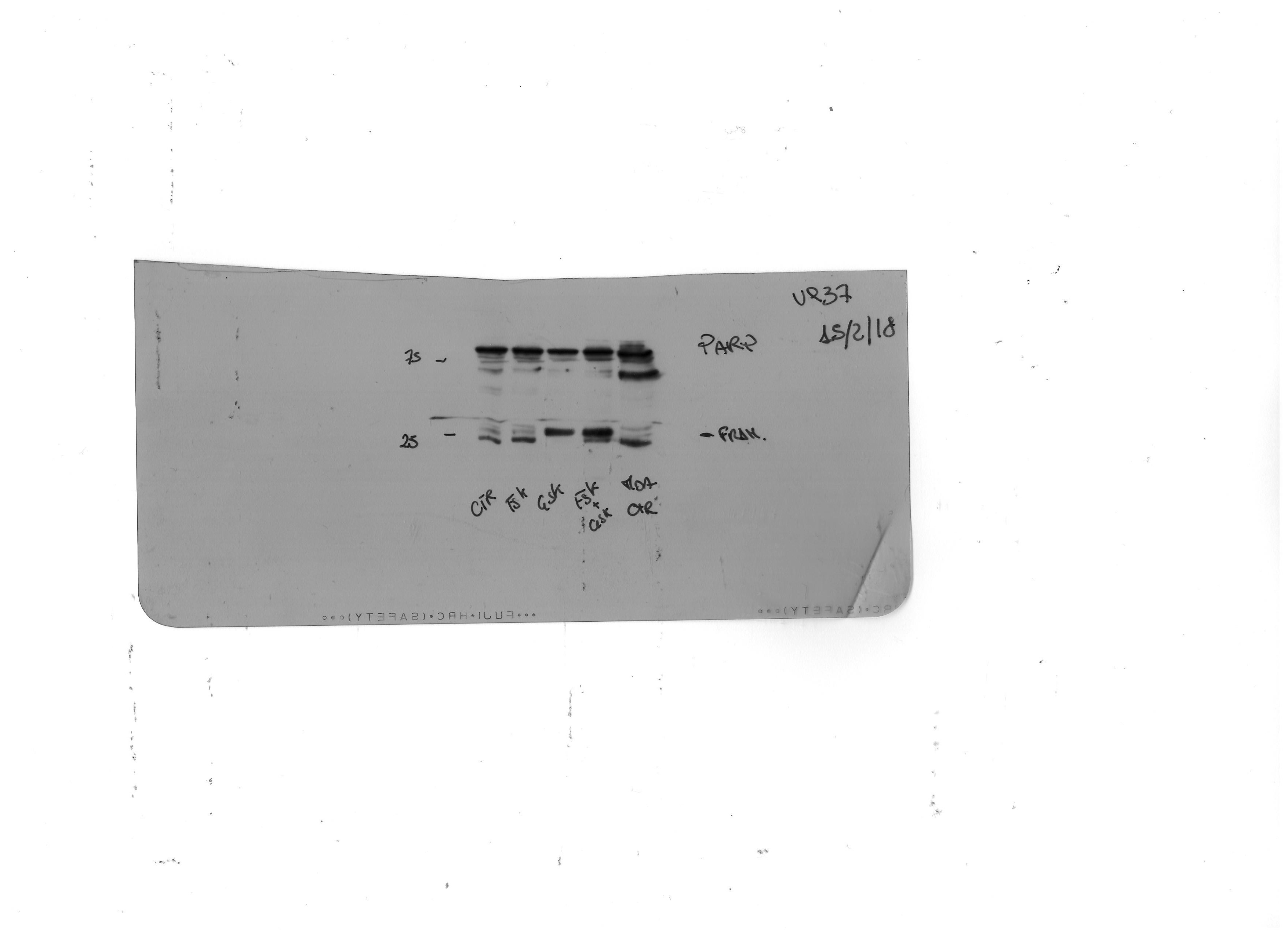

Supplement: Supplementary file 3 [file Data_Sheet_1.ZIP › 382492 _supp images/parp 15.02.jpg]
